# Respiratory afflictions during hairdressing jobs: case history and clinical evaluation of a large symptomatic case series

**DOI:** 10.1186/s12995-022-00351-5

**Published:** 2022-05-23

**Authors:** Julia Hiller, Annette Greiner, Hans Drexler

**Affiliations:** Institute and Outpatient Clinic of Occupational, Social and Environmental Medicine, Friedrich-Alexander-Universität Erlangen-Nürnberg, Henkestr. 9-11, 91054 Erlangen, Germany

**Keywords:** Occupational health, Asthma, Rhinitis, Respiratory system, Ammonium persulfate, Hairdresser

## Abstract

**Objectives:**

Respiratory symptoms at work are common among hairdressers. Various working materials, most notably bleaching ingredients such as ammonium persulfate, have been made responsible. The objective of this study is to achieve a better understanding of work-related respiratory symptoms of hairdressers by describing common features in a large affected collective.

**Methods:**

One hundred forty-eight hairdressers with respiratory symptoms at work presenting between 2012 and 2019 were consecutively included in a case series. Anamnestic and diagnostic data including pulmonary function and allergy testing were retrospectively compiled from records and analysed. Additionally, cases were categorised in five groups with respect to occupational causation certainty.

**Results:**

30% of the predominantly female collective had changed jobs or were on longer sick-leave. Besides respiratory symptoms, 10% also reported contact urticaria to blonde dyes. In 60% an obstructive airway disease was confirmed. A specific hypersensitivity reaction to ammonium persulfate was found in 15%. Group 1 with a proven immunological occupational causation showed significantly lower age (*p* < 0.001) and tenure time (*p* = 0.001), higher sensitization rates against environmental allergens as well as a higher total IgE (*p* = 0.015), compared to group 4 (obstructive airway disease, specific occupational causation unlikely).

**Conclusions:**

This case series contributes to a better characterization of work-related respiratory symptoms in hairdressing as one of the largest examined collectives of symptomatic hairdressers. Ammonium persulfate as the most common specific cause showed signs of a type-I-like hypersensitivity reaction with typical risk factors for atopy. Prick testing is recommended in all symptomatic cases. However, a specific occupational causation often cannot be proved.

**Supplementary Information:**

The online version contains supplementary material available at 10.1186/s12995-022-00351-5.

## Introduction

A high prevalence of upper and lower respiratory symptoms and increased asthma risks in hairdressers have been repeatedly reported [1–7]. Besides questionnaire-based reports on symptoms also multiple case reports or smaller case series describe asthma related to bleaching products or persulfate salts [8–13]. Occasionally, atypical late airway reactions [14, 15] and cases with airway reactions to other substances used in hairdressing such as paraphenylenediamine [10], hair spray [16, 17], formaldehyde from straightening products [18], wheat protein from styling products [19], or henna [20] have been described.

In two older larger collectives of 47 and 55 hairdressers with work-related respiratory diseases specific inhalation challenges and skin tests identified persulfate salts and bleaching powder as the major causative agents in 22-45% of the cases [21, 22]. However, a few cases with other causative agents (permanent hair dyes, latex, paraphenylenediamine) were also confirmed by specific inhalation challenges [21]. Another symptomatic hairdresser collective was identified by telephone interview, 109 cases met the criteria for a suspected occupational disease and were further clinically examined (skin tests, lung function, in some cases specific provocation) [3]. However, the collective included respiratory and skin affected cases. In seven cases asthma and/or allergic rhinitis was diagnosed as an occupational disease and in all cases ammonium persulfate was considered a causative agent [3]. Nevertheless, the exact underlying pathomechanism for those work-related reactions remain unclear.

Especially for persulfate salts an immunologic mechanism has often been suggested but so far was not definitely and comprehensively demonstrated [23]. Considering reports of immediate airway reactions and other type-I-allergy-like reactions such as urticaria or anaphylaxis [10, 24] as well as delayed-type reactions [15, 25], multiple varying mechanisms could be conceivable.

Due to those uncertainties and many reports being rather timeworn, it is important to shed further light on common case features and clinical results of those currently affected.

## Methods

### Study design and data extraction

This retrospective descriptive study is based on a consecutive case series of 148 hairdressers with current or previous respiratory symptoms at work, who have been referred to an outpatient department for evaluation of an occupational influence between 2012 and 2019. Most cases were seen in a specialized clinic for work-related respiratory problems implemented in 2012.

The main study objective was to systematically characterize the symptomatic collective of hairdressers with respect to their case features and clinical results to identify common characteristics. Secondly, we aimed at a case categorization concerning the certainty of a specific occupational causation of the respiratory symptoms.

All medical case documentations at hand were reviewed and the compiled data from anamnesis and available medical examinations (e.g. pulmonary function and allergy testing) were extracted anonymously. The study design by retrospective chart review, data collection and processing were approved by the local ethics committee.

### Diagnostics

Details on equipment and materials used are reported in the additional file 1.

Blood panel including IgE was carried out by an accredited medical laboratory and evaluated by the age and sex-adjusted reference values.

Skin prick tests (SPT) were done with various environmental inhalants (available preparations changed over time) and a freshly prepared 5% test solution for ammonium persulfate (AP) by solving 0.5 g AP with 10 ml Aqua dest. In some cases, a self-prepared henna solution and prick-to-prick with latex glove were tested as well. The tests were read off after 20 min and rated as positive if the wheal reached at least 3 mm [26]. Cases with abnormal findings in the positive and negative controls were critically assessed and mostly excluded from further SPT evaluation.

Lung function was evaluated by bodyplethysmography, with airway obstruction diagnosed when elevated airway resistance (sRtot ≥1.2 kPa*s) and/or FEV1/FCV <5th percentile (Z-Score < − 1.645) (with sufficient collaboration in forced manoeuvre) was found [27–29]. When those criteria were not fully fulfilled, but the breathing loops showed a beginning kinking the case was rated as borderline obstruction.

To determine bronchial hyperreactivity (BH) methacholine challenge testing (MCT) was performed with a five-step provocation protocol up to a cumulative dose of 0.471 mg methacholine [30]. The standard positivity criteria (doubling of sRtot and simultaneous increase to ≥2.0 kPa*s or FEV1-fall > 20%) were used [30]. For clinical purposes, a negative MCT is assumed when no positivity criteria are reached until cumulatively 0.471 mg methacholine. However, in occupational medicine a cumulative provocation dose of methacholine ≤0.3 mg is regarded as the limit [30]. Therefore, we rated MCT as positive when the positivity criteria were reached until a cumulative methacholine dose of ≤0.3 mg. Additionally, cases with antiobstructive premedication (active during the consultation) that either fulfilled the positivity criteria at a higher cumulative provocation dose of up to 0.471 mg methacholine or stayed marginally below the positivity criteria with methacholine ≤0.3 mg were also considered indicative of a BH. Patients without a premedication and a positive reaction at a cumulative methacholine dose of > 0.3 mg were rated as borderline. All patients who had used short or long-acting bronchodilatators on the day of the consultation or corticosteroids within a week of the consultation were considered to receive an antiobstructive medication active during lung function testing as inhaled corticosteroids can contribute to the decrease of airway obstruction through their anti-inflammatory properties.

Compliant with the guidelines [29] an obstructive ventilation disorder (OVD) was assumed when either current obstruction or BH (together with symptoms consistent with asthma) were found, in borderline cases a suspected OVD was assumed. Some cases remained unclear when lung function data were incomplete, due to contraindications for or early termination of MCT.

In cases with strong anamnestic hints for a specific trigger but inconclusive diagnostic results from SPT and lung function testing specific inhalation challenges (SIC) with hair dyes were performed. The patients were asked to perform hairdyeing tasks with their problematic work substances in a provocation chamber for 30 min. Baseline pulmonary function (including MCT) was performed before the start. Subsequent lung function testing was performed for up to 6 hours after provocation. Afterwards another MCT was performed. The SIC was rated as positive according to aforementioned criteria for MCT [27, 30] or when a significant increase in BH after the provocation could be objectified.

Certain patient features were combined for a shortened atopic diathesis score (SADS) of maximum 7 points: 1 point each for a personal history of atopic dermatitis, allergic rhinitis and allergic asthma to environmental allergens as well as positive family history of atopic diseases (2 pt. when respiratory and skin diseases were reported in the family) and total IgE of > 150 U/ml (2 pt. for > 400 U/ml).

### Data analysis

The primary outcome measure of our study was the characterization of a symptomatic hairdressers’ collective with respect to demographics, medical data/anamnesis (including underlying conditions, atopy, smoking status, family anamnesis, environmental exposures), symptom anamnesis and course, working history and conditions as well as medical results in pulmonary function and prick testing. Secondary was a case categorization according to the certainty of a specific occupational causation and a comparison of the above-mentioned criteria among those categories.

The case categorization constituted a composite measure taking anamnestic and diagnostic data together. This evaluation was done by two researchers reviewing the aggregated data independently before ascribing the case to a category (Table 1). Afterwards, case allocation was compared and in case of discrepancy discussed until consensus was reached. Categorization was performed for *n* = 147 as in one case hairdressing was not the focus of the medical consultation.

**Table 1:** Group allocation scheme for certainty of specific occupational causation* among *n =* 147 hairdressers

Group: Specific occupational causation	1: Confirmed (*n =* 24)	2: Likely, but not conclusively proven (*n =* 11)	3: Unclear (*n =* 25)	4: Unlikely (*n =* 66)	5: No occupat. Causation (*n =* 21)
**Airway disease verified**	**Yes**	**Yes**	**Yes**	**Yes**	**No**
I. Obstructive ventilation disease confirmed	*n =* 5	*n =* 1	*n =* 5	*n =* 23	–
II. Work-related rhinoconjunctivitis	*n =* 9	*n =* 3	*n =* 7	*n =* 19	–
III. both	*n =* 10	*n =* 7	*n =* 13	*n =* 24	–
**Immediate-type-like hypersensitivity reaction to AP or hair dyes**	**Confirmed**	**Likely, but not unequivocally confirmed**	**Unlikely**	**No**	**No**
IV. “Positive” reaction to AP or blonde dye in skin (prick) testing	*n =* 21 with CR^a^	*n =* 1 CR unsure^a^	*n =* 1 CR unlikely^a^	–	–
V. “Questionable” reaction to AP or blonde dye in skin (prick) testing	*n =* 1 with CR^a^ (positive SIC)	*n =* 4^b^	*n =* 3 CR unlikely^a^ (1x neg. SIC)	*n =* 3 without CR^a^(2x neg. SIC)	*n =* 1 without CR^a^ (neg. SIC)
VI. “Negative” skin prick test to AP/blonde dye	–	*n =* 2^b, c^	*n =* 14^d^	*n =* 63^e^	*n =* 20
VII. Rating of skin testing for immediate-type-like reactions to AP / blonde dye: “not appraisable”	*n =* 2 but positive SIC	*n =* 3^b^	*n =* 6^d^	–	–
VIII. No skin prick test to AP/blonde dye conducted	–	*n =* 1^b^	*n =* 1	–	–

*Specific occupational causation primarily based on affirmation status of an airway disease and verifiability of a specific hypersensitivity reaction (*AP* Ammonium persulfate; *CR* Clinical relevance; *SIC* Specific inhalation challenge), for more details on the reasoning of the hypersensitivity reaction please also see Additional file 2

^a^ Clinical relevance based on anamnesis (suitable, exposure-dependent / disputable / incongruous / no symptoms at contact) and, if performed, SIC-result (negative SIC = CR unlikely or excluded)

^b^ No SIC performed, but with clear anamnestic indications for an occupational causation (such as urticaria at skin contact to hair/blonde dyes, hints at anaphylactic reactions to hair dyes, peakflow protocol showing workplace-related deterioration)

^c^ One case with positive prick test to henna and contact urticaria to p-phenylendiamine and henna and recurrent angioedema and respiratory symptoms after dark dyes; other case with contact urticaria to blonde dye and positive peakflow protocol

^d^ Some, but less conclusive indications for a substance-specific occupational causation

^e^ Anamnestically no / few indications for a substance-specific occupational causation

Statistical analyses were conducted with IBM SPSS Statistics and OriginLab Origin® 2019. Due to the explorative case series study setup no sample size estimation was made and primarily descriptive analyses were performed. Variance analysis among subgroups were performed with Kruskal-Wallis, when necessary post-hoc tests were performed with Bonferroni correction. *P*-value was set at < 0.05 for significance.

## Results

### Collective characterization

Some selected characteristics of the collective are summarized in Table 2 (demographic, general medical data and environmental exposure), Table 3 (specifics of work-related symptoms) and Table 4 (diagnostic evaluation), a more comprehensive overview of the personal and medical characteristics and the working conditions is presented as an additional online file (see Additional file 3). Aspects that need to be further addressed are followed up in the text with additional information.

**Table 2:** Selected personal characteristics of study collective (*n =* 148) concerning demographics, general medical data and environmental exposure

Characteristics				N	%
Female sex				136	91.9
Smoking status *(missing data* *n* = *2)*					
Never smoker				59	39.9
Ex-smoker				60	40.5
Current smoker				27	18.2
Private pet contact				58	39.2
Positive family anamnesis for atopic diseases				56	37.8
Type I sensitization to environmental inhalation allergens (positive SPT during consultation or previous external findings)				92	62.6
Known allergic rhinoconjunctivitis to ubiquitous inhalation allergens				58	39.1
Known allergic asthma to ubiquitous inhalation allergens				13	8.8
**Characteristics**	**Median**	**Mean**	**SD**	**N**	**%**
Total IgE [U/ml] *(missing data n* = *5)*	27.0	92.9	185.4		
Fraction with > 100 U/ml				30	20.3
Shortened atopic diathesis score [points] (max. 7)	1.0	1.22	1.2		
Fraction with ≥2 points				46	31.1

**Table 3:** Selected characteristics of work-related symptoms and specific anamnesis of study collective (*n =* 148)

Characteristics	Range	Median	Mean	SD
Age at initial symptoms [years] *(missing data* *n* = *3)*	15 – 67	34.0	34.0	12.4
Hairdresser tenure at initial symptoms [years] *(missing data* *n* = *11)*	0 – 44.5	12.5	15.0	11.9
Duration of symptoms at work [years] *(missing data* *n* = *9)*	0.3 – 36	3.0	5.5	7.2
**Categorial variables**			**N**	**%**
Change of job or on sick-leave ≥2 month			45	30.4
Work-related nasal symptoms			92	62.2
Work-related cough and/or sore throat			123	83.1
Work-related dyspnea, wheezing or chest tightness			137	92.6
Progression of upper to lower airway symptoms (only nasal afflictions in the beginning, but cough or dyspnoea later on)			25	16.9
Exclusively upper airways symptoms the whole time			3	2.0
Most common specific workplace triggers named:				
Hair dyes in general			123	83.1
Blonde dyes			120	81.1
No particular workplace triggers named: stay in saloon in general problematic			8	5.4
Dust, fume, vapor or odour named as unspecific (extra-professional) triggers for airway symptoms			101	68.2
Reported seasonal fluctuation of symptoms			36	24.3
History of work-related hand eczema			54	36.5
Known contact sensitization to hairdressers’ substance(s) overall (incl. AP)			32	21.6
Known contact sensitization to AP			12	8.1
Work-related urticaria to hair dyes			14	9.5

**Table 4:** Selected diagnostic results and medical evaluation of study collective (*n =* 148)

Characteristics	N	%
Methacholine challenge test (performed* in)	*114*	*77.0*
Positive	50	33.8
Borderline	7	4.7
Negative	46	31.1
Discontinued early, not appraisable	11	7.4
*Not performed due to contradictions like relevant initial obstruction, massive hypertension, pregnancy or patient refusal.		
Skin prick test at consultation against AP or own blonde dye *(performed in)*	144	97.3
*Not appraisable due to insufficient controls*	*9*	*6.1*
Positive or questionable reaction	30	20.3
Previous external skin tests against AP or own blonde dye *(performed in)*	9	6.1
Positive or questionable reaction	8	5.4
Skin prick test at consultation against henna *(performed in)*	22	14.9
Positive or questionable reaction	2	1.4
Specific inhalation challenge (SIC) *(performed in)*	9	6.1
SIC positive for AP or blonde dye	3	2.0
Specific occupational causation for obstructive airway disease confirmed (via clinically relevant hypersensitivity reaction to AP/blonde dye or positive SIC)	24	16.2

Concerning private pet contacts (reported by 39%), cats (*n =* 26) and dogs (*n =* 30) were the most commonly named pet types (multiple answers possible). From a medical standpoint a positive reaction in the SPT to the own pet type might be of further importance and was present in 19% of the cases with private pet contacts (corresponding to 7% overall).

Besides pets as important extra-professional exposures other ubiquitous allergens might influence airway symptoms and diseases as well. In this context, sensitization to environmental inhalation allergens is also relevant. From the SPT performed during consultation and other external findings such sensitization could be assumed in 63% of the collective. Grass/grains and tree pollens were the most common positive allergens. Based on the reports of a known allergic rhinoconjunctivitis or asthma to environmental allergens, anamnesis suggested that at least 66% of those sensitizations were clinically relevant. However, the shortened atopic diathesis score overall was indicative of a rather low atopic diathesis prevalence.

**Fig. 1:**
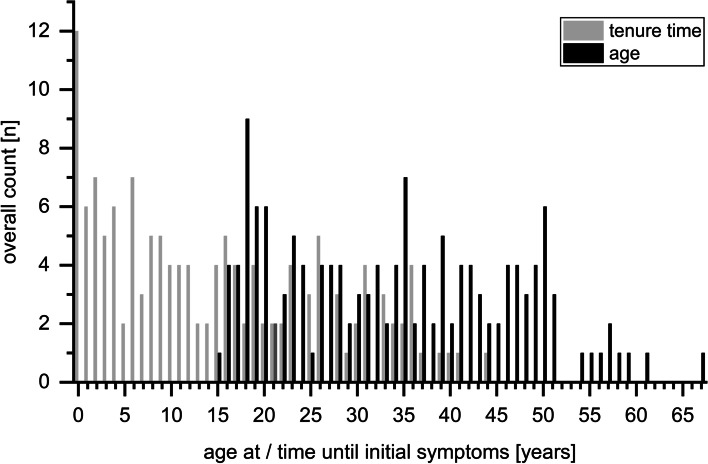
Tenure as a hairdresser (*n =* 137; grey) and age (*n =* 145; black) at initial symptoms at work

**Fig. 2:**
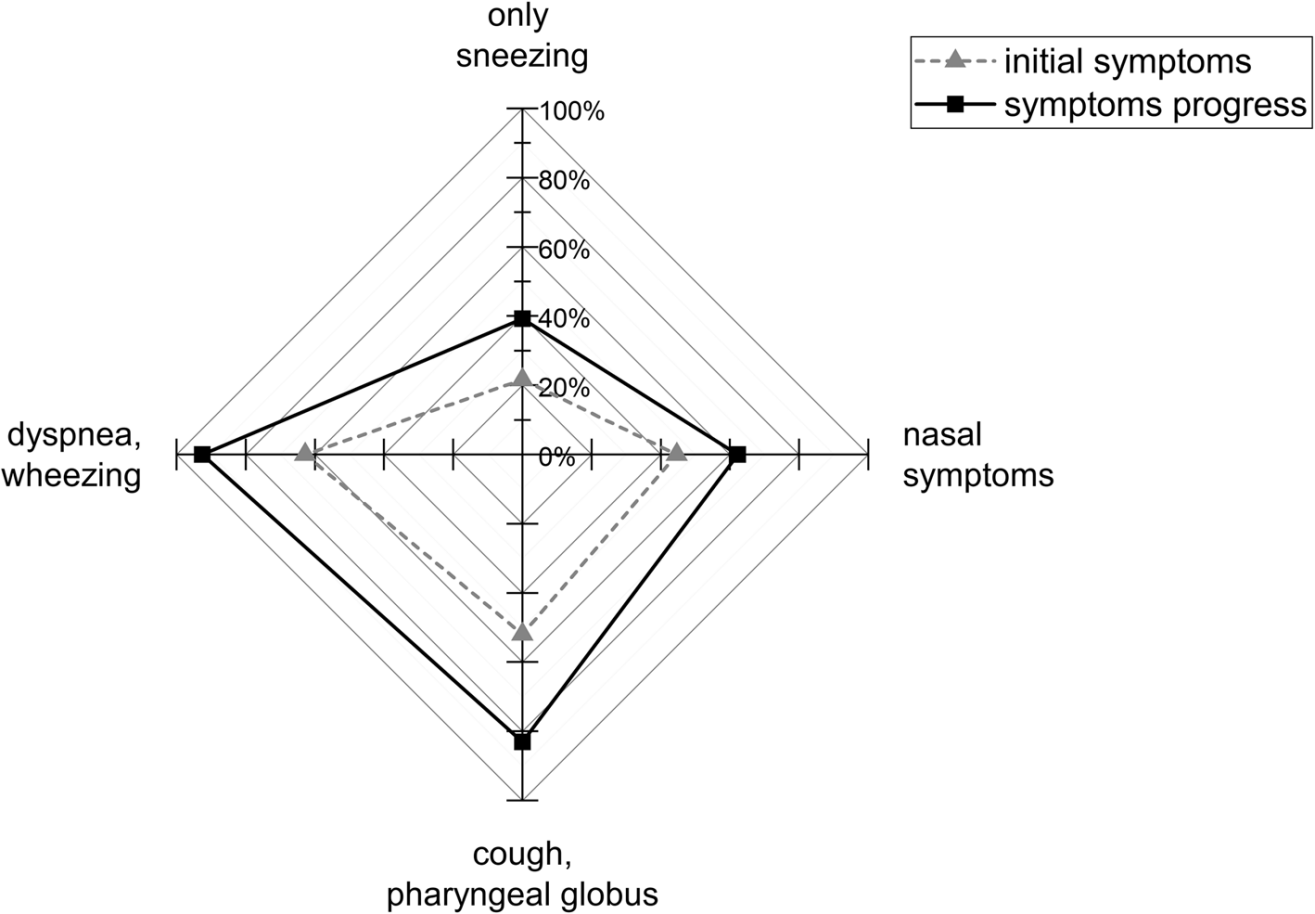
Expansion of indicated initial symptoms (dotted line) during course of the disease (black line)

Regarding the specifics of the work-related anamnesis the following aspects shall be further pointed out. While representing the full spectrum of the working population, tenure time as a hairdresser and age at initial symptoms were not normally distributed but rather clearly left-skewed respectively suggestive of a bimodal age distribution (Fig. 1). Many cases had also described an expansion of initial work-related symptoms over time (Fig. 2). Especially a progression from pure upper to additional lower airway symptoms (as seen in 17%) can be of high medical relevance. Only very seldom no progress towards the lower airways occurred. While most cases named hairdressing specific triggers (certain substances or stay in the saloon in general) for their airway symptoms, two cases described no hairdressing specific exposures as triggers at all, but rather an aggravation by work stress and smell of customers’ perfumes. Usually workplace symptoms started within 10 min of exposure (73% of cases with available data (*n =* 79)). When a seasonal fluctuation of symptoms was reported, such aggravation was attributed to weather-conditions (warm-humid, cool-damp), reduced ventilation in winter or seasonal allergens.

Besides airway symptoms some cases also described an immediate reaction in form of a contact urticaria to hair/blonde dyes or AP, mostly concomitant with the first airway symptoms. Other skin alterations were described as well, especially hand eczema and known contact sensitizations to hairdressers’ substances. From those cases with a contact sensitization against AP, three cases described also an urticarial reaction to blonde dyes.

With respect to the diagnostics and medical evaluation it seems important to point out, that despite the synopsis of current lung function data (bodyplethysmography and MCT) from the consultation only allowed the diagnosis of an OVD in 49%, overall an obstructive lower airway disease could be confirmed in 60%. For this, accountable previous lung function results were also considered for the final diagnosis, when available, to account for possibly concealing effects like premedication (the antiobstructive medication active during lung function testing included bronchodilatators in 34%) or exposure abstention.

For evaluation of a specific immediate type reaction to workplace substances SPT were performed where possible (2x contradictions: pregnancy and severe previous anaphylactic reaction not suitable for ambulant testing; 1x patient refusal). Almost all cases were tested with AP or a test solution of their own blonde dye (in one case the reason for presentation was not the former hairdressing job), but not all tests produced appraisable results due to insufficient controls. Furthermore, henna was tested in a smaller subset due to exposure anamnesis. Previous skin tests to AP or blonde dye from other institutions/consultations were considered as well. Overall, positive or questionable skin reactions to AP or blonde dye were present in *n =* 37 cases (one with positive reaction in SPT on-site and previous positive test). To evaluate the clinical relevance of those skin reactions, exposure-specific anamnesis and additional SIC were also considered. By this, clinical relevance of the skin test reactions to AP or blonde dyes could be established for *n =* 22 cases (15%). Furthermore, two cases without appraisable SPT to AP (due to urticaria factitia) had a positive SIC (for more details see case-by-case breakdown in additional file 2). Therefore, a specific occupational causation for an obstructive airway disease was overall confirmed in 16% (*n =* 24), although in the two last-named cases this could not be surely attributable to a specific hypersensitivity reaction to hairdressers' substances (invalid SPT).

For henna SPT gave two positive reactions. One without clinical relevance and one indicative of a clinically relevant specific hypersensitivity (contact urticaria to previous henna tattoo and in unprotected skin contact to clients’ self-henna-dyed hair), however it could not be considered the primary causative agent for the work-related respiratory symptoms as no henna was utilised in the saloon directly (see also additional file 2).

Latex was also pricked in some cases and gave five positive or questionable SPT reactions but was not rated as relevant for the respiratory symptoms in any case due to anamnesis.

### Categorisation and subgroup comparison

**Fig. 3:**
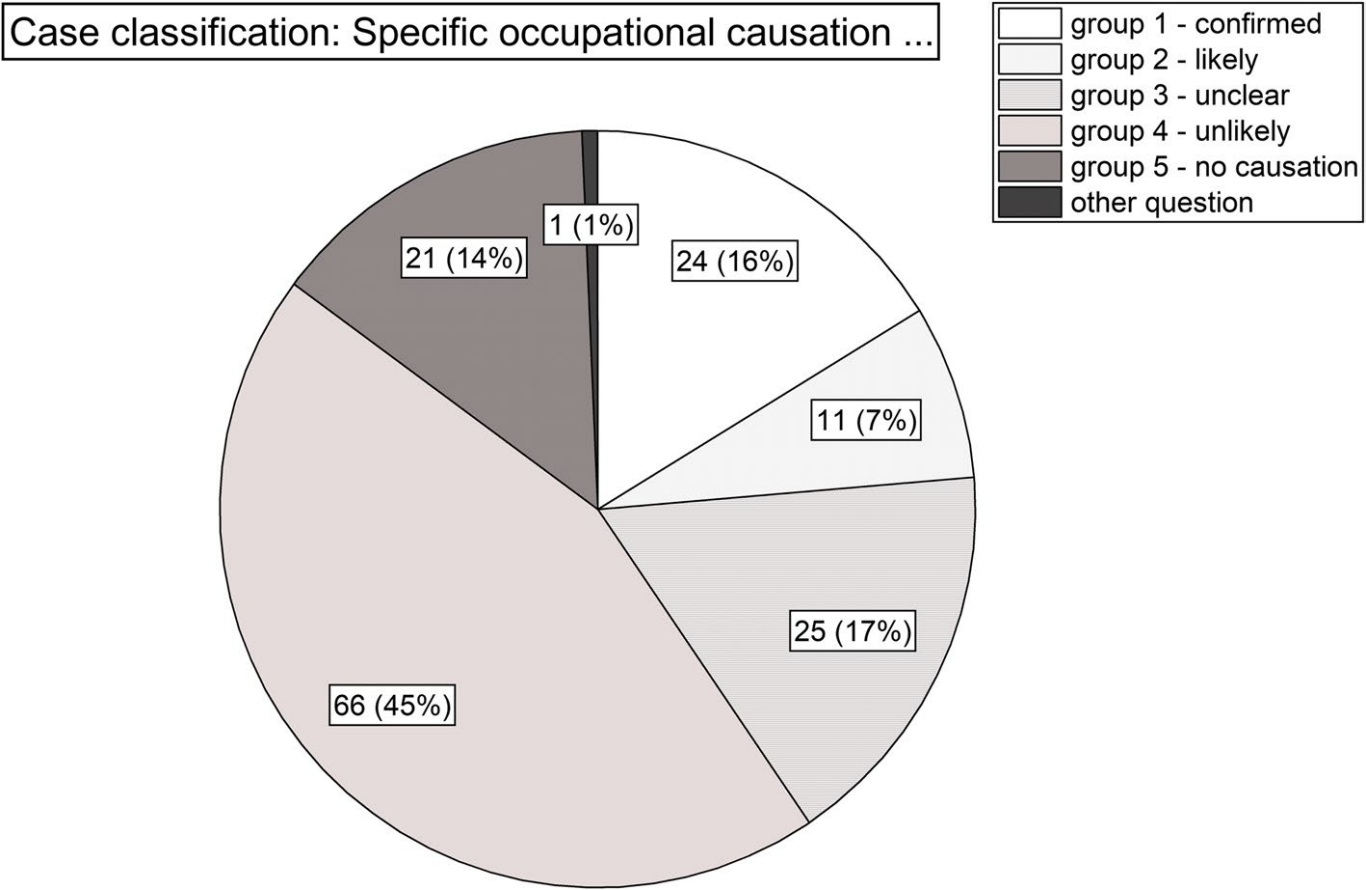
Case classification according to certainty of a specific occupational causation of obstructive airway disease

Figure 3 shows the overall distribution of case categories among the collective. While in about 45% a specific occupational causation from hairdressing was unlikely, it was unclear in 17% and likely or proven in almost a quarter of the collective. Due to no objectifiable respiratory diagnosis no occupational causation could be discussed for 14%.

Among group 1 and 2 (confirmed or likely causation) cases with a rhinitis were slightly more common than cases with an obstructive lower airway disease (= widely referred to as asthma) as shown in Table 1. However, even more common was a combination of both affected systems (lower and upper airways). Applying the most severe criteria an occupational asthma was asserted in the 15 cases with an obstructive lower airway disease and confirmed occupational causation (10%) and an occupational rhinitis was asserted for 13% (*n =* 19 cases), but with some significant overlap between both disease entities (for details see Table 1). Comparison of patients’ characteristics among the defined categories 1 to 5 for substance-specific causation revealed several interesting aspects (full breakdown for the five categories included in the supplementary table in Additional file 3). For additional visual illustration of the magnitude of subgroup variations an orientating heat map of selected parameters is also included in the Additional file 4.

While age and tenure time at initial symptoms are naturally associated (as shown by a supplementary figure, see Additional file 5), both were considerably lower in group 1 than in groups 2 to 5 (see supplementary table in Additional file 3). Analysis of variance confirmed a significant difference among groups (age (*n =* 144): H = 17.182, *p* = 0.002; tenure (*n =* 136): H = 15.387, *p* = 0.004). Post-hoc tests revealed group 1 vs. 4 for age (*p* < 0.001) and tenure (*p* = 0.001) and group 1 vs. 5 (*p* = 0.046) for age as differing. Accordingly, the bimodal distribution of age and left-skewed tenure time among the overall collective (Fig. 1) is largely an effect based on group 1, as shown by group-wise depiction of those parameters (as shown by a supplementary Fig. S2, see Additional file 6).

Contrary smoking behaviour was comparably distributed among groups and variance analysis for pack years (*n =* 51 data) also showed no differences (H = 7.261, *p* = 0.123).

Interestingly, group 5 not only missed an OVD diagnosis and rhinoconjunctival symptoms (by definition) but also showed fewer hand eczema. In contrast, group 1 and 2 had an increased prevalence of known contact sensitization against hairdressers’ substances and especially AP. Furthermore, they more often complained of urticaria to blonde dyes and AP.

Also features concerning atopy showed significant differences among groups. Group 1 showed a higher total IgE average and more cases with increased IgE (> 100 U/mL) while the opposite was true for groups 4 and 5. Variance analysis confirmed significant group differences for IgE (H = 17.209, *p* = 0.002; *n =* 142) with the post-hoc tests showing group 1 differing from group 4 (*p* = 0.015) and 5 (*p* = 0.038). Accordingly, group 1 showed the highest average SADS and group 5 the lowest. Here post-hoc analysis of the significant group variances (H = 14.416, *p* = 0.006; *n =* 147) pointed to group 5 as the differing one (group 5 vs. 1: *p* = 0.003; group 5 vs. 3: *p* = 0.042; group 5 vs. 4: *p* = 0.035). This was further underlined by the high percentage of cases with 0 points in the SADS for group 5. Furthermore, the prevalence of allergic rhinoconjunctivitis, positive SPT and type-I-sensitization to environmental inhalation allergens was much less common in group 5, but tended to be increased in group 1 and to lesser extent group 3.

However, some subgroup abnormalities, especially concerning nature of workplace symptoms, lung function data, AP SPT and final diagnoses, were primarily caused by category definition (see Table 1).

## Discussion

Hairdressing is associated with increased adverse respiratory outcomes (lung function decline, respiratory symptoms at workplace and in general) [5, 7]. In the herein reported symptomatic collective almost all cases experienced symptoms of the lower respiratory tract and in 60% an obstructive lower airway disease was confirmed. The ongoing relevance of hairdresser asthma is demonstrated by presentation of 148 cases in 8 years. Especially the high percentage of job changers or patients on longer sick-leave emphasizes the high socio-economic significance and need of further clarification of respiratory symptoms in those symptomatic hairdressers. The case series also represents a large group of systemically AP prick tested individuals (*n =* 134 appraisable tests) with persulfate salt exposure, showing comparable positivity rates (~ 15%) as reported in other exposed industry sectors [31].

Comparisons with previous reports on respiratory symptoms in hairdressers are limited by differentiating methodologies, being rarely based on merely symptomatic collectives. Bearing those differences in mind and accounting for influences from special subgroups studied (e.g. apprentices), demographics and tenure time were mostly in line with the expectations for hairdressers from the full spectrum of the working population [1, 2, 4, 5, 21, 22]. While current smokers were found to a lesser extent than often described [3, 21, 22, 32], the prevalence was comparable to current data from the German female population (18.6%) [33]. Our data on total IgE (mean and elevated rate (> 100 U/ml)) were comparable to older surveys among hairdressers [32].

The previously described collectives of symptomatic hairdressers date back up to 20 years and also applied partly different diagnostic approaches [3, 21, 22]. Schwaiblmair et al. [22] reported 22% positive SIC and 24% positive SPT to bleaching powder. However, no specifics on the bleaching powder or its components were given. For assumption of a specific immunological hypersensitivity reaction persulfate salts are the relevant component to consider, but other bleaching powder components might provoke unspecific irritative reactions when those preparations are tested as a whole which could possibly cause false-positive results. The group definitions introduced by the authors (I: with or II: without asthmatic symptoms) were also not quite comparable, still a higher rate of atopic features (environmental sensitization, atopic diseases) showed in group I while smoking status and prevalence of rhinitic symptoms did not differ significantly. This trend is mostly confirmed by our results. The reported rate of BH (58%) was also higher than in our collective. Leino et al. [3] found only seven positive SPT reactions to persulfates (in 107 tests), no current obstruction in spirometry in respiratory-effected cases and lower rates of proven BH (19%). However, the overall rate of confirmed occupational diseases in the collective was also small and cases with only skin symptoms were also included. Nonetheless, a previously diagnosed atopic disease also increased the risk for an occupational skin or respiratory disease 3-fold. Moscato et al. [21] reported comparable data of BH and obstructive airway diagnoses but based their evaluation of occupational causation primarily on SIC with nebulisation of an AP solution. However, the relation of the nebulised concentration to real-life workplace airborne exposure remains unclear. Moreover, SPT to AP were only performed in two-thirds of the patients with positive SIC and showed no positives. Still persulfate salts were deemed responsible for a confirmed occupational asthma in 88%, comparable to what we saw in group 1 (92%). Interestingly, patch tests against AP were positive in 25% of the SIC-positive patients [21], something we also saw in comparable magnitude (29%) in our group 1 (confirmed occupational causation; positive SPT to AP).

While the reported overall prevalence of contact sensitization to AP (8%) in our collective was two to three times lower than that of systematically patch-tested hairdressers with a work-related dermatitis (95%CI: 15.4-21.9%), it was still three to six times higher than in non-hairdressing female patients (with hair cosmetics suspected as dermatitis cause) (95%CI: 1.3-2.8%) [34]. However, as only about a third of our collective had a history of occupational dermatitis and not nearly all patients had received or reported a patch test this reference is a bit misleading, probably underestimating the real prevalence of a concomitant contact sensitization. Compared to contact sensitizations to hairdressers’ substances overall, AP sensitization was prevalent in 38%. More so, 25% of our patients with AP contact sensitization also described urticarial reactions to blonde dyes. Higher rates of positive patch tests than expected might be a coincidence due to high rates of simultaneously present contact allergies caused by the workplace exposure, but could also possibly be falsified by a misinterpreted otherwise caused skin reaction such as contact urticaria underscoring the postulation of a specific immediate-type-like hypersensitivity reaction.

Appraising our case categorization one must know that the German occupational disease ordinance allows the recognition of an occupational disease for obstructive allergic airway reactions in the form of asthma and rhinopathy. Therefore, group 1 and 2 (proven or likely specific occupational causation) included cases without a proven OVD of the lower airways but work-related rhinopathy, as a possible precursor for asthma. Contrary, cases with neither an OVD nor reported occupational rhinitis could not be considered for an occupational causation (group 5). Furthermore, it is undisputable that airborne chemicals can also have irritant effects and induce irritant asthma [35]. General occupational exposure to dust, fumes, vapors and aerosols has also been shown to be associated with increased bronchial reactivity [36], possibly a precursor for respiratory symptoms or asthma. Therefore, it is possible, that especially some of the cases in group 3 and 4 are caused by general irritative occupational exposures in hairdressing. However, it is hardly possible to mark-off such irritant-induced afflictions from extra-professional caused OVD or to objectify them conclusively by clinical tests as a clearly attributable specific occupational causation. Moreover, work-related asthma also encompasses work-exacerbated asthma where extra-professional induced asthma deteriorates due to workplace-related but rather unspecific exposures [37]. While a negative occupational influence should not be denied, such cases would likely experience increased airway symptoms at other unfavorable workplaces outside of hairdressing as well. This notion especially relates to group 4, where despite a high prevalence of confirmed OVD (71%) a specific occupational causation was unlikely. However, a hairdressing profession might be very unfavorable in some of those cases.

While some special features of group 1 revealed through subgroup comparison (e.g. younger age, higher prevalence of environmental sensitization/atopy, urticaria to AP) support the assumption of a hypersensitivity reaction against AP reminiscent of a type-I-allergy and show important risk factors for it, the interpretation of some other subgroup variations is limited due to differences in lung function data, AP SPT and final diagnoses being caused by group definitions. Other limitations might be caused by the retrospective analysis of medical records. Therefore, recall, reporting and observer bias should be kept in mind. Especially underreporting of certain primarily anamnestic features cannot be completely excluded. Furthermore, in some cases with questionable, insufficient or negative findings in lung function diagnostics an OVD might be disguised by active antiobstructive premedication or condition regression during exposure abstention. Partly, this can be counteracted through consideration of previous findings, however those were not always available or of acceptable quality. Lastly, the different and sometimes small subgroup sizes should be kept in mind when comparing group characteristics, especially parameters with low overall prevalence should be interpreted with some caution. Despite their rather explorative nature, they are still valuable for the overall picture.

In conclusion, this case series contributes to a better characterization of work-related respiratory symptoms in hairdressing and is to our knowledge one of the largest clinically examined collective of symptomatic hairdressers to date, therefore constituting an important data collection to identify risk factors and similarity among cases. Still, the clarification of causes for work-related airway symptoms often remains problematic with possible irritative effects of hairdressers’ substances and 14% even missing any evaluable respiratory diagnosis. Contrary, AP was the most common (15%) identifiable specific cause and showed multiple signs reminiscent of a type-I-allergy (as described above and seen in the detailed case-by-case descriptions in additional file 2). Furthermore, AP as a confirmed specific cause of airway diseases (group 1) was associated with risk factors for atopy and typical symptoms of high-molecular-weight agents, although AP has a low-molecular weight [38]. Those findings constitute important novel insights into the possible pathological effects of AP as a very recent literature review [39] found that the majority of retrieved studies did “not support a type I allergic reaction as a pattern of respiratory responses to” persulfate salts as most publications reported negative prick tests. Therefore, we postulate a specific immediate-type-like hypersensitivity reaction as one route of mechanism for AP and recommend uniform prick testing with AP in all symptomatic cases. Special attention should be given to atopic individuals who seem to be at a higher risk.

## Supplementary Information

**Additional file 1. **Equipment and materials used (Manufacturer’s names and addresses).

**Additional file 2. **Case-by-case evaluation of clinical relevance regarding hypersensitivity reactions to hairdresser’s substances (Supplementary Table).

**Additional file 3. **Comprehensive collective characteristics (Supplementary Table).

**Additional file 4. **Heat map – visual illustration of the magnitude of subgroup variations.

**Additional file 5. **Association between age and tenure time at initial symptoms (supplementary figure).

**Additional file 6. **Age and tenure time at initial symptoms overall and broken down by group-wise depiction of those parameters among case classification for specific immunological occupational causation (supplementary figure). 

## Data Availability

All data relevant to the study are included in the article or uploaded as additional information, no further raw data are available due to confidentiality reasons concerning medical patient data.
