# Supplementary material for: Respiratory afflictions during hairdressing jobs: case history and clinical evaluation of a large symptomatic case series
Source: J Occup Med Toxicol. 2022 May 23;17:10. doi: 10.1186/s12995-022-00351-5 (PMC9125837; doi:10.1186/s12995-022-00351-5)
Supplement: Supplementary file 1 — Additional file 1. Equipment and materials used (Manufacturer’s names and addresses). [file 12995_2022_351_MOESM1_ESM.pdf]

Additional file 1: Equipment and materials used (Manufacturer's names and addresses)

- Commercially available common environmental inhalants from Allergopharma® GmbH & Co KG (Reinbeck, Germany), Bencard® Allergie GmbH (Munich, Germany), and Laboratoris Leti® (Barcelona, Spain).
- Ammonium persulfate ≥ 98% from Sigma-Aldrich® (Saint Louis, MO, USA).
- Aqua dest. 10 ml (Aqua mini-plasco® connect) from B. Braun Melsungen AG (Melsungen, Germany).
- Lung function testing with bodyplethysmography und unspecific inhalation test was performed with the MasterScreen Body/Diff ®Jäger from Vyaire medical (Hoechberg, Germany).
- For the metacholine challenge the aerosolized particles were generated by the APS pro Nebulizer Head ®Jäger from CareFusion/Vyaire medical (Hoechberg, Germany) and provocation was performed with the reservoir method PARI Provocation Test® II (PARI GmbH, Starnberg, Germany). For this a 3.2% metacholine solution prepared by the hospital pharmacy of the university hospital Erlangen-Nürnberg was used.
- Statistical analyses were conducted with IBM® SPSS® Statistics (Version 25) (Armonk, NY, United States) and OriginLab Origin® (Northampton, Massachussets, United States).
