# Supplementary material for: Respiratory afflictions during hairdressing jobs: case history and clinical evaluation of a large symptomatic case series
Source: J Occup Med Toxicol. 2022 May 23;17:10. doi: 10.1186/s12995-022-00351-5 (PMC9125837; doi:10.1186/s12995-022-00351-5)
Supplement: Supplementary file 2 — Additional file 2. Case-by-case evaluation of clinical relevance regarding hypersensitivity reactions to hairdresser’s substances (Supplementary Table). [file 12995_2022_351_MOESM2_ESM.pdf]

<u>Additional file 2:</u> Case-by-case evaluation of clinical relevance regarding hypersensitivity reactions to hairdresser's substances (Supplementary Table)

Individual evaluation of cases with either positive specific inhalation challenge (SIC) or positive or questionable skin test reactions (wheal ≥ 1mm) to specific hairdresser's substances (Ammonium persulfate (AP) and henna) during consultation (from n=32 valid skin prick tests (SPT) without urticarial factitia) and from previous (external) tests (n=8) and their case-related clinical relevance appraisal. Skin test readings are given with *wheal diameter / reddening diameter in mm* (where available).

**SPT at consultation**	**Previous skin test (type, results and quality)**	**Clinical anamnesis**	**SIC to blonde dye**	**Clinically relevant**
Henna ≥ 3 mm wheal (N=2)				
3/3	/	No reactions to henna contact known	/	No
3/3	/	Known contact urticaria to personal henna tattoo and in contact with henna-dyed hair from customers; no direct utilisation of henna in own saloon. Additionally, respiratory symptoms and urticaria to dark hair dyes; known contact allergy to p-Phenylendiamine	SIC advised in stationary setting	**Yes**
SPT to ammonium persulfate or blonde dye not appraisable (Urticaria factitia) (N=2)				
3/35 but with urticaria factitia (NaCl 5/50)	/	Rhinoconjunctivitis to aeroallergens since childhood, over time additional workplace-dependent cough and dyspnea, especially to blonde dyes and dyes with H_2O_2; SPT not appraisable due to urticaria factitia; PEF protocol showing workplace-related deterioration; despite exposure reduction progression of symptoms in follow-up => SIC performed: positive with significant FEV1 reduce	SIC positive	**Yes**
AP n.t. own blonde dyes negative (0/2 & 0/3)	SPT: urticaria factitia<br>AP 1% = +<br>AP 5% = -<br>(allergy ward of dermatology department)	Rhinoconjunctivitis, cough and urticaria at work to blonde dyes;<br>External SPT to AP not appraisable due to urticaria factitia => SIC performed	SIC: rhinitic symptoms positive	**Yes**
Questionable skin test reaction (Test quality and reactions not appraisable) to ammonium persulfate or blonde dye (N=2)				
n.t. (no SPT) Medically supervised SPT in	Self-experiment at home: Blonde dye dissolved in water applied to arm	Initially recurrent infects, over time general unspecified airway symptoms; later sweating, vertigo, fatigue and circulation problems; recently nasal obstipation, ichting eyes, cough and dyspnea at work with hair dyes, blonde dyes and permanent wave;	/	Unclear, not apprais-able

stationary setting was recommended but refused by the patient	Anamnestically 3h later urticaria at the contact site and in face and tachycardia, diarrhoe, swelling of the tongue and dyspnea described by patient	Self-experiment at home with description of urticaria and possibly anaphylactic symptoms 3h later => Immediate presentation in emergency room, where no pathological findings were objectified => not appraisable		
0/0 negative under 2 month abstention	Previous SPT:<br>special blonde (+++)<br>blonde dye (++)<br>hair spray (+)<br>permanent wave (++)<br>(ENT physician)<br>⇨ Not fully appraisable, disputable unspecific irritation	Immediate rhinoconjunctivitis in contact with blonde dyes, dyes in general, permanent wave; over time also cough and dyspnea; additionally, ichting, reddening and pustules after skin contact to blonde dye; symptoms as a bystander and deterioration during own work<br>Previous SPT (from ENT physician) positive for blonde dyes, hair spray and permanent wave, therefore previous reaction to all tested work-material (unspecific irritation cannot be ruled out); but principally anamnesis fitting, SPT not reproducable under short exposure abstention	/	Possible, causation likely
Questionable skin test reaction (wheal ≥ 1 mm and < 3mm) to ammonium persulfate or blonde dye (N=11)				
0/0 negative after 18 month abstention	SPT:<br>AP + H2O2 = 2/3<br>(department of occupational medicine)	Cough, dyspnea, eye symptoms and nausea in contact with blonde dyes and hair dyes; Since termination of hairdressing no improvement questionable reaction to previous combined SPT testing of AP and H2O2 => SIC performed	SIC negative	No
0/4 negative under continued inhalative exposure	Allergy pass from dermatologist describing SPT: (H=5, K=0)<br>1. Silk Lift Condition Creme = erythema<br>2. Silk Lift Control = 0<br>3. Blonde dye = erythema + swelling<br>All 3 mixed = gigantic wheal	Initially generalized urticaria after blonde dyeing of the own hair, since then no further skin contact, but rhinoconjunctivitis and dyspnea to blonde dyes;<br>Previous SPT performance and documentation of questionable quality, evaluation unclear => SIC performed	SIC negative	No
2/2	/	Rhinitis and dyspnea only at workplace reported => SIC performed	SIC negative	No
2/2	/	Rhinoconjunctivitis at workplace => SIC performed	SIC negative	No

1/4	/	Usage of blonde dye powder is tolerated without problems; (exertional) dyspnea to various substances and smells	/	No
1/5	/	Cough and dyspnea to hair dyes when ammonia is released; nasal obstruction only to hair spray	/	No
2/2	/	No symptoms during contact to blonde dyes reported, SPT to own blonde dyes negative	/	No
2/2	/	Cough and dyspnea to hair dyes, permanent wave and hair spray; previous problems also during occupational soldering	/	Unclear
2/2	/	Sneezing and dyspnea to hair dyes, permanent wave and other hairdressing substances; better after saloon renovation	/	Unclear
2/2	/	Throat tickling and cough to hair dyes, blonde dyes and H_2O_2	/	Unclear
2/20	/	Rhinoconjunctival symptoms to blonde dyes; improvement since abstention, SPT performed 43 month after last contact => SIC performed	SIC: nasal symptoms positive	**Yes**
Positive skin reaction (wheal ≥ 3 mm) to ammonium persulfate or blonde dye (N=23)				
3/3	/	**No respiratory afflictions specific to blonde dyes documented;** General exertional dyspnea and dyspnea to smells (general and in saloon)	/	No
3/6	/	Only occasional nasal obstruction in saloon reported, other rhinoconjunctival symptoms not in saloon; inspiratory constriction in throat reported to hair dyes, no exspiratory symptoms; no obstruction or BH in lung function testing	/	Unclear
0/0 negative under 34 month abstention	SPT<br>blonde dye powder = 10/13<br>AP = 10/13<br>(from allergo-dermatologic expert report)	Rhinoconjunctivitis and asthmatic symptoms strictly to blonde dye contact; Since abstention no problems anymore; additional hand eczema	/	**Yes**
0/0 negative after 10 month abstention	Scratch test:<br>blonde dye = +++<br>blonde dye powder = ++++<br>(performed under exposure)	Rhinoconjunctivtis, cough and dyspnea in contact with blonde dyes; additional skin vesicles; Since abstention significant improvement	/	**Yes**

3/3	/	Shortly after start of apprenticeship rhinoconjunctivitis, thoracic tightness, face reddening, wheezing and vertigo within 20 min of colouring, especially to blonde dyes	/	**Yes**
3/3	/	General rhinoconjunctivitis and cough, over time rhinitis, cough and dyspnea pronounced at work, especially when colouring; since abstention for 8 month significant improvement	/	**Yes**
3/7	/	Rhinoconjunctivitis and over time dyspnea to hair dyes (blonde dyes the worst); skin reddening after direct contact to blonde dyes	/	**Yes**
3/10	/	Striktly work-related sneezing, cough and dyspnea immediately when colouring; termination of hairdressing 2 month ago, since then recovery	/	**Yes**
3/17	/	Sneezing and dyspnea at saloon, symptoms especially pronounced in contact with blonde dyes; PEF (peakflow) protocol showing workplace-related deterioration	/	**Yes**
3/20	/	Cough and dyspnea after contact to hair dyes and hair spray; no rhinoconjunctivitis; besides SPT to AP, also SPT with own blonde dye products positive => SIC was recommended but performed	/	**Yes**
4/6	/	Rhinoconjunctivitis and globus sensation within minutes of hair dye contact, in the evening cough and wheezing; worst symptoms with brighter colours; severe hand eczema occurred concomitant	/	**Yes**
4/8	/	Initially ichting and redding of the skin after direct skin contact to blonde dye, over time rhinitis and dyspnea during dye blending; no improvement since termination of job two years ago; also known contact allergy to AP	/	**Yes**
4/15	/	Dry cough, known rhinoconjunctivitis and asthma to ubiquitous inhalation allergens; more cough at work; externally SIC performed (not formally positive, however borderline reaction); additionally, PEF protocol showing workplace-related deterioration<br>⇨ Evaluation as newly developed hypersensitivity reaction against AP	Former SIC: Some reaction, not formally positive	**Yes**

4/30	/	General exertional dyspnea, with worsening at workplace and additional thoracic tightness and cough, especially one hour after blonde dyes; improvement during longer sick-leave	/	**Yes**
5/15	/	Dyspnea and cough immediately at contact to blonde and brighter hair dyes, additional rhinitic symptoms; over time also urticaria on face and hands during hairdyeing	/	**Yes**
5/23	Previous SPT anam-nestically positive for blonde dyes (no protocol available)	Rhinoconjunctivitis initially only to blonde dyes and highlights at work; later also thoracic tightness and globus sensation; over time also other triggers for symptoms; additionally known contact allergy to AP	**/**	**Yes**
5/25	/	Initially rhinoconjunctivitis, later thoracic tightness and dyspnea at work; especially to blonde dyes; hardly any symptoms without work	**/**	**Yes**
5/40	/	Immediate sneezing at blonde dye contact, over time also dyspnea; PEF protocol showing workplace-related deterioration	/	**Yes**
6/13	/	Initially cough and dyspnea over work day, later rhinoconjunctivitis and dyspnea immediately during colouring; first only at blonde dyes, later also to other colours and as a bystander; no problems without working; additionally hand eczema and known contact allergy to AP; also skin reddening and swelling in the face in the saloon	/	**Yes**
6/20	/	Urticaria and dyspnea after skin contact to blonde dye with stationary monitoring afterwards; since then no further own blonde dyeing, but rhinoconjunctivitis and dyspnea as a bystander; other hair dyes without problems; additionally hand eczema	/	**Yes**
8/8	/	Dyspnea especially from blonde dyes; known atopic dermatitis	/	**Yes**
8/40	/	Initially rhinitis, over course also cough and dyspnea, one time redding of the face; now immediate respiratory symptoms at work, especially from blonde dyes and other dyes; PEF protocol with work-related detoriation; additionally hand eczema	/	**Yes**
10/38	/	During apprenticeship first globus sensation, over time also rhinoconjunctivitis and dyspnea, especially to blonde dyes (immediately), also cough at hair spray and smells; additionally over time urticaria to skin contact with blonde dyes; also hand eczema	/	**Yes**
