# Supplementary material for: Respiratory afflictions during hairdressing jobs: case history and clinical evaluation of a large symptomatic case series
Source: J Occup Med Toxicol. 2022 May 23;17:10. doi: 10.1186/s12995-022-00351-5 (PMC9125837; doi:10.1186/s12995-022-00351-5)
Supplement: Supplementary file 3 — Additional file 3. Comprehensive collective characteristics (Supplementary Table). [file 12995_2022_351_MOESM3_ESM.pdf]

Additional file 3: Comprehensive collective characteristics (Supplementary Table)

Personal and medical characteristics of a symptomatic hairdressers' collective (n=148) with a history of respiratory symptoms at work for overall collective (*left column*) and broken down by five categories *(right columns)* rating certainty of occupational causation for n=147

Parameter: Overall collective (n=148)	*Group 1 (n=24) confirmed*	*Group 2 (n=11) likely*	*Group 3 (n=25) unclear*	*Group 4 (n=66) unlikely*	*Group 5 (n=21) no causation*
**Demographics, general medical data and environmental exposure**					
**Sex:** 91.9% women	95.8% women	81.8% women	96.0% women	90.09% women	90.4% women
**Age at consultation [years]:** M 39.0; Ø 39.7 ± 13.7<br>R: 16-78	M 25.5; Ø 28.6<br>R: 19-49	M 42.0; Ø 42.6<br>R: 28-65	M 39.0; Ø 38.4<br>R: 21-58	M 47.5; Ø 43.9<br>R: 16-78	M 39.0; Ø 39.9<br>R: 18-69
**BMI [kg/m²]:** M 24.3; Ø 25.5 ± 5.6<br>R: 17.5-52.1 (comparable among sexes)<br>➢ **BMI > 30kg/m²:** 16.2% (n=24)	M 23.1; Ø 24.0<br>R: 17.8-34.1<br>➢ 8.3% (n=2)	M 25.2; Ø 25.7<br>R: 21.3-29.9<br>➢ 0% (n=0)	M 24.4; Ø 26.3<br>R: 18.1-46.7<br>➢ 20.0% (n=5)	M 24.7; Ø 26.2<br>R: 17.5-52.1<br>➢ 21.2% (n=14)	M 23.3; Ø 24.0<br>R: 17.5-32.9<br>➢ 21.4% (n=3)
**Smoking history**<br>➢ **Never smoker:** 39.9% (n=59)<br>➢ **Ex-smoker:** 40.5% (n=60)<br>o **Pack Years:** M 4.0; Ø 9.8; R: 0.4-66 (a.d. n=29)<br>➢ **Current smoker:** 18.2% (n=27)<br>o **Pack Years:** M 6.5; Ø 11.8; R: 1-43 (a.d. n=22)<br>➢ **Missing data:** 1.4% (n=2)	 50.0% (n=12)<br>33.3% (n=8)<br>n.c.<br>16.7% (n=4)<br>n.c.<br>-	 27.3% (n=3)<br>45.5% (n=5)<br>n.c.<br>27.3% (n=3)<br>n.c.<br>-	 56.0% (n=14)<br>28.0% (n=7)<br>n.c.<br>12.0% (n=3)<br>n.c.<br>4.0% (n=1)	 33.3% (n=22)<br>45.5% (n=30)<br>n.c.<br>19.7% (n=13)<br>n.c.<br>1.5% (n=1)	 38.1% (n=8)<br>42.8% (n=9)<br>n.c.<br>19.0% (n=4)<br>n.c.<br>-
**Antiobstructive medication:** 74.3% (n=110)<br>➢ **permanent:** 58.8% (n=87)<br>➢ **active* during lung function testing**: 58.1% (n=86)<br>** use of bronchodilatators within the last 24 hours or corticosteroids within the last 7 days*	62.5% (n=15)<br>37.5% (n=9)<br>41.7% (n=10)	81.8% (n=9)<br>72.7% (n=8)<br>72.7% (n=8)	84.0% (n=21)<br>64.0% (n=16)<br>60.0% (n=15)	75.8% (n=50)<br>63.6% (n=42)<br>60.6% (n=40)	66.7% (n=14)<br>57.1% (n=12)<br>61.9% (n=13)
**Private pet contact:** 39.2% (n=58) (n=128 a.d.)<br>➢ **positive SPT to own pet type(s):** 18.9% (n=11) of pet contacts	50.0% (n=12)<br>58.3% (n=7)	36.4% (n=4)<br>-	28.0% (n=7)<br>28.6% (n=2) + 1x questionable	37.9% (n=25)<br>8.0% (n=2) + 1x questionable	47.6% (n=10)<br>-
**Occupational anamnesis / Working conditions**					
**Duration of symptoms at work [years]:** (n=139 a.d.)	(n=23 a.d.)	(n=10 a.d.)		(n=59 a.d.)	

Parameter: Overall collective (n=148)	*Group 1 (n=24) confirmed*	*Group 2 (n=11) likely*	*Group 3 (n=25) unclear*	*Group 4 (n=66) unlikely*	*Group 5 (n=21) no causation*
M 3.0; Ø 5.5 ± 7.2<br>R: 0.25-36;	M 2.5; Ø 3.5<br>R: 0.25-25	M 2.0; Ø 10.0<br>R: 0.5-31.5	M 2.0; Ø 4.3<br>R: 0.5-19.5	M 3.5; Ø 6.5<br>R: 0.5-36	M 2.0; Ø 4.0<br>R: 0.25-25
**Age at initial symptoms at work [years]:** (n=145 a.d.)<br>M 34.0; Ø 34.0 ± 12.4<br>R: 15-67	(n=23 a.d.)<br>M 21.0; Ø 24.9<br>R: 18-46	M 35.0; Ø 32.8<br>R: 16.5-44	M 35.0; Ø 34.1<br>R: 18-55	(n=64 a.d.)<br>M 39.5; Ø 37.2<br>R: 15-61	M 35.0; Ø 35.7<br>R: 16-67
**Hairdresser tenure at initial symptoms [years]:** (n=137 a.d.) M 12.5; Ø 15.0 ± 11.9<br>R: 0-44.5	(n=23 a.d.)<br>M 4.5; Ø 6.6<br>R: 0-22.5	(n=10 a.d.)<br>M 16.3; Ø 13.0<br>R: 0.5-26	M 14.5; Ø 15.3<br>R: 0.5-37	(n=57 a.d.)<br>M 16.0; Ø 18.2<br>R: 0-44.5	M 15.0; Ø 16.4<br>R: 0-40.5
**Change of job:** 23.6% (n=35)<br>➢ **Time since [month]**: M 7.0; Ø 17.8; R: 0.5-192	37.5% (n=9)<br>n.c.	9.1% (n=1)<br>n.c.	16.0% (n=4)<br>n.c.	24.2% (n=16)<br>n.c.	19.0% (n=4)<br>n.c.
**On sick-leave ≥ 2 month:** 6.8% (n=10)<br>➢ **Duration [month]**: M 4; Ø 4.9; R: 2-14	12.5% (n=3)<br>n.c.	9.1% (n=1)<br>n.c.	4.0% (n=1)<br>n.c.	7.6% (n=5)<br>n.c.	-<br>n.c.
**Currently self-employed hairdresser:** 23.0% (n=34)	20.8% (n=5)	27.3% (n=3)	28.0% (n=7)	19.7% (n=13)	28.6% (n=6)
**Full-time work (≥ 35h/week):** 40.5% (n=60) overall<br>(59% of a.d. (n=102))	45.8% (n=11)	27.3% (n=3)	48.0% (n=12)	43.9% (n=29)	19.0% (n=4)
**History of mold at workplace:** 6.1% (n=9)<br>➢ **currently still present:** 2.7% (n=4)	4.2% (n=1)<br>4.2% (n=1)	-<br>-	4.0% (n=1)<br>4.0% (n=1)	9.1% (n=6)<br>3.0% (n=2)	4.8% (n=1)<br>-
**Number of customer seats in service [n]**: (n=86 a.d.)<br>M 7; Ø 7.1 ± 3.9<br>R: 1-30	(n=16 a.d.)<br>M 8; Ø 7.1<br>R: 2-12	(n=7 a.d.)<br>M 8; Ø 8.1<br>R: 4-13	(n=11 a.d.)<br>M 6; Ø 6.6<br>R: 1-12	(n=42 a.d.)<br>M 7; Ø 7.6<br>R: 1-30	(n=10 a.d.)<br>M 4; Ø 4.5<br>R: 2-
**Saloon size [m²]:** (a.d. n=54)<br>M 60; Ø 61; R: 10-120	n.c.	n.c.	n.c.	n.c.	n.c.
**Portion of working time spend colouring [%]** (a.d. n=40)<br>M 50; Ø 43; R: 0-75	n.c.	n.c.	n.c.	n.c.	n.c.
**Separated room for dye preparing:** 26.4% (n=39) overall<br>(49% of a.d. (n=80))	25.0% (n=6)	18.2% (n=2)	32.0% (n=8)	30.3% (n=20)	14.3% (n=3)
**Use of gloves at work:** 70.2% (n=104) overall<br>(97% of a.d. (n=107))	83.3% (n=20)	81.8% (n=9)	60.0% (n=15)	71.2% (n=47)	61.9% (n=13)
**Regular fresh air at saloon:** 61.5% (n=91) overall<br>(85% of a.d. (n=107))	75.0% (n=18)	63.6% (n=7)	60.0% (n=15)	54.5% (n=36)	71.4% (n=15)
**Technical ventilation / air conditioning:** 23.0% (n=34)	12.5% (n=3)	36.4% (n=4)	16.0% (n=4)	27.3% (n=18)	23.8% (n=5)

Parameter: Overall collective (n=148)	*Group 1 (n=24) confirmed*	*Group 2 (n=11) likely*	*Group 3 (n=25) unclear*	*Group 4 (n=66) unlikely*	*Group 5 (n=21) no causation*
(37% of a.d. (n=92))					
**Specific case anamnesis**					
**Airway symptoms at work (multiple answers possible)**					
➢ **Upper airways**					
➢ **pure conjunctival (without nasal affection):** initially 2.7% (n=4) / *over time (ot) 6.8% (n=10)*	4.2% (n=1) / *ot 4.2% (n=1)*	- / -	4.0% (n=1) / *ot 8.0% (n=2)*	- / -	9.5% (n=2) / *ot 19.0% (n=4)*
➢ **nasal (with/without conjunctival affection):** initially 44.6% (n=66) / *ot 62.2% (n=92)*	62.5% (n=15) / *ot 79.2% (n=19)*	72.7% (n=8) / *ot 90.9% (n=10)*	60.0% (n=15) / *ot 80.0% (n=20)*	42.4% (n=28) / *ot 65.2% (n=43)*	- / -
➢ **sneezing:** initially 21.6% (n=32) / *ot 39.2% (n=58)*	45.8% (n=11) / *ot 70.8% (n=17)*	18.2% (n=2) / *ot 36.4% (n=4)*	16.0% (n=4) / *ot 40.0% (n=10)*	22.7% (n=15) / *ot 40.9% (n=27)*	- / -
➢ **other rhinitic symptoms (excl. sneezing):** initially 41.9% (n=62) / *ot 55.4% (n=82)*	54.2% (n=13) / *ot 62.5% (n=15)*	72.7% (n=8) / *ot 90.9% (n=10)*	60.0% (n=15) / *ot 72.0% (n=18)*	39.4% (n=26) / *ot 59.1% (n=39)*	- / -
➢ **Lower airways**					
➢ **cough and/or sore throat:** initially 52.0% (n=77) / *ot 83.1% (n=123)*	50.0% (n=12) / *ot 87.5% (n=21)*	45.5% (n=5) / *ot 81.8% (n=9)*	32.0% (n=8) / *ot 76.0% (n=19)*	54.5% (n=36) / *ot 84.8% (n=56)*	76.2% (n=16) / *ot 85.7% (n=18)*
➢ **dyspnoea, wheezing or chest tightness**: initially 62.8% (n=93) / *ot 92.6% (n=137)*	66.7% (n=16) / *ot 100% (n=24)*	54.5% (n=6) / *ot 100% (n=11)*	64.0% (n=16) / *ot 96.0% (n=24)*	60.6% (n=40) / *ot 86.4% (n=57)*	66.7% (n=14) / *ot 95.2% (n=20)*
**Symptoms progression from exclusive upper airways initially to lower airways over time:** 16.9% (n=25)	25.0%(n=6)	27.3% (n=3)	24.0% (n=6)	13.6% (n=9)	4.8% (n=1)
**Exclusively upper airway symptoms the whole time:** 2.0% (n=3)	-	-	12.0% (n=3)	-	-
**Start with concurrent bronchial infect:** 13.5% (n=20)	12.5% (n=3)	18.2% (n=2)	4.0% (n=1)	13.6% (n=9)	n=5 (24%)
**Seasonal influence / fluctuation:** 24.3% (n=36)	37.5% (n=9)	9.1% (n=1)	36.0% (n=9)	19.7% (n=13)	19.0% (n=4)
**Latency between daily exposure start and airway symptoms:** n=79 a.d.	(n=16 a.d.)	(n=7 a.d.)	(n=15 a.d.)	(n=34 a.d.)	(n=7 a.d.)
➢ **immediate within 10 min:** 73.4% (n=58) of a.d.	68.8% (n=11) a.d.	42.9% (n=3) a.d.	73.3% (n=11) a.d.	82.4% (n=28) a.d.	71.4% (n=5) a.d.
➢ **more 1h:** 7.6% (n=6) of a.d.	12.5% (n=2) a.d.	14.3% (n=1) a.d.	4.9% (n=1) a.d.	2.9% (n=1) a.d.	14.2% (n=1) a.d.
**Improvement only during vacation/longer time off work:** 23.0% (n=34) overall (30% of a.d. (n=112))	16.7% (n=4)	18.2% (n=2)	12.0% (n=3)	27.3% (n=18)	33.3% (n=7)
**Symptoms improvement during weekends:** 48.6% (n=72) overall (71% of a.d. (n=101))	58.3% (n=14)	72.7% (n=8)	76.0% (n=19)	33.3% (n=22)	42.8% (n=9)

Parameter: Overall collective (n=148)	*Group 1 (n=24) confirmed*	*Group 2 (n=11) likely*	*Group 3 (n=25) unclear*	*Group 4 (n=66) unlikely*	*Group 5 (n=21) no causation*
**Workplace trigger stay in saloon in general:** 5.4% (n=8)	4.2% (n=1)	-	-	7.6% (n=5)	9.5% (n=2)
**Specific workplace triggers named (multiple answers possible):** 93.2% (n=138)<br>➢ **hair dyes in general**: 83.1% (n=123)<br>➢ **blonde dyes:** 81.1% (n=120)<br>➢ **hair spray:** 60.1% (n=89)<br>➢ **permanent wave**: 35.8% (n=53)	95.8% (n=23)<br>79.2% (n=19)<br>95.8% (n=23)<br>37.5% (n=9)<br>25.0% (n=6)	100% (n=11)<br>90.9% (n=10)<br>100% (n=11)<br>54.5% (n=6)<br>54.5% (n=6)	100% (n=25)<br>100% (n=25)<br>76.0% (n=19)<br>76.0% (n=19)<br>28.0% (n=7)	90.9% (n=60)<br>78.8% (n=52)<br>78.8% (n=52)<br>69.7% (n=46)<br>37.8% (n=25)	85.7% (n=18)<br>76.2% (n=16)<br>71.4% (n=15)<br>42.9% (n=9)<br>42.9% (n=9)
**Work-related urticaria in contact with hair/blonde dyes or AP**: 9.5% (n=14)<br>➢ **urticarial skin afflictions already at symptoms start**: 6.8% (n=10)	25.0% (n=6)<br>20.8% (n=5)	45.5% (n=5)<br>27.3% (n=3)	8.0% (n=2)<br>4.0% (n=1)	1.5% (n=1)<br>1.5% (n=1)	-<br>-
**History of work-related hand eczema**: 36.5% (n=54)<br>➢ **duration [years]**: M 2.8; Ø 7.1; R: 0.5-27; (a.d. n=20)<br>➢ **current hand eczema at consultation**: 8.1% (n=12)	45.8% (n=11)<br>n.c.<br>8.3% (n=2)	36.4% (n=4)<br>n.c.<br>9.1% (n=1)	40.0% (n=10)<br>n.c.<br>4.0% (n=1)	37.8% (n=25)<br>n.c.<br>12.1% (n=8)	14.3% (n=3)<br>n.c.<br>-
**Known contact sensitization to hairdressers' substance(s) overall:** 21.6% (n=32)<br>➢ **known contact sensitization to AP**: 8.1% (n=12)<br>➢ **in cases with hand eczema**: 33.3% (n=18) of hand eczema	50.0% (n=12)<br>29.1% (n=7)<br>63.6% (n=7) of hand eczema	45.5% (n=5)<br>9.1% (n=1)<br>75.0% (n=3) of hand eczema	20.0% (n=5)<br>4.0% (n=1)<br>20.0% (n=2) of hand eczema	12.1% (n=8)<br>4.5% (n=3)<br>24.0% (n=6) of hand eczema	9.5% (n=2)<br>-<br>-
**Unspecific triggers for airway symptoms (multiple answer possible)**<br>➢ **dust, fume, vapor, odour:** 68.2% (n=101)<br>➢ **stress in general:** 24.3% (n=36)<br>➢ **aggravation by airway infect:** 20.3% (n=30)<br>➢ **ubiquitous inhalation allergens**: 11.5% (n=17)	75.0% (n=18)<br>20.8% (n=5)<br>20.8% (n=5)<br>25.0% (n=6)	72.7% (n=8)<br>36.4% (n=4)<br>9.1% (n=1)<br>-	60.0% (n=15)<br>28.0% (n=7)<br>8.0% (n=2)<br>20.0% (n=5)	71.2% (n=47)<br>22.7% (n=15)<br>24.2% (n=16)<br>6.1% (n=4)	61.9% (n=13)<br>19.0% (n=4)<br>28.6% (n=6)<br>9.5% (n=2)
**Selected preexisting conditions**					
**Known hypertonus**: 14.9% (n=22)	-	27.3% (n=3)	16.0% (n=4)	18.2% (n=12)	14.3% (n=3)
**Recurrent or chronic sinusitis or s/p sinus surgery**: 24.3% (n=36)	20.8% (n=5)	18.2% (n=2)	24.0% (n=6)	28.8% (n=19)	19.0% (n=4)
**Nasal polyps (or s/p polyp surgery):** 10.8% (n=16)	8.3% (n=2)	-	8.0% (n=2)	16.7% (n=11)	4.8% (n=1)

Parameter: Overall collective (n=148)	*Group 1 (n=24) confirmed*	*Group 2 (n=11) likely*	*Group 3 (n=25) unclear*	*Group 4 (n=66) unlikely*	*Group 5 (n=21) no causation*
**Abnormalities of the nasal concha or septum or s/p surgery:** 9.4% (n=14)	-	-	8.0% (n=2)	16.7% (n=11)	4.8% (n=1)
**Reflux oesophagitis:** 24.3% (n=36)	16.7% (n=4)	27.3% (n=3)	28.0% (n=7)	28.8% (n=19)	14.3% (n=3)
**Known thyroid issue (s/p, hyper- or hypofunction):** 21.6% (n=32)	20.8% (n=5)	18.2% (n=2)	28.0% (n=7)	24.2% (n=16)	9.5% (n=2)
**Known or suspected sleep apneoa:** 2.7% (n=4)	-	9.1% (n=1)	4.0% (n=1)	3.0% (n=2)	-
**Allergic rhinoconjunctivitis to ubiquitous inhalation allergens:** 39.1% (n=58)<br>➢ **duration [years]:** M 14; Ø 16.1; R: 1-38 (a.d. n=22)	41.7% (n=10)<br>n.c.	45.5% (n=5)<br>n.c.	48.0% (n=12)<br>n.c.	40.9% (n=27)<br>n.c.	19.0% (n=4)<br>n.c.
**Allergic asthma bronchiale to ubiquitous inhalation allergens:** 8.8% (n=13)<br>➢ **duration [years]:** M 10.5; Ø 12.4; R: 2-25 (a.d. n=6)	12.5% (n=3)<br>n.c.	9.1% (n=1)<br>n.c.	16.0% (n=4)<br>n.c.	7.6% (n=5)<br>n.c.	-<br>n.c.
**(History of) atopic dermatitis:** 8.8% (n=13); (n=4 only as child)	20.8% (n=5)	-	8.0% (n=2)	9.1% (n=6)	-
**Atopic anamnesis**					
**Positive family anamnesis for atopic diseases (in 1° relatives):** 37.8% (n=56) (n=135 a.d.)	54.2% (n=13)	45.5% (n=5)	32.0% (n=8)	37.9% (n=25)	23.8% (n=5)
**SADS (max. 7 points)**: M 1.0; Ø 1.22 ± 1.2<br>R: 0-6	M 1.5; Ø 1.75<br>R: 0-6	M 1.0; Ø 1.27<br>R: 0-4	M 1.0; Ø 1.48<br>R: 0-4	M 1.0; Ø 1.17<br>R: 0-5	M 0.0; Ø 0.52<br>R: 0-4
➢ **0 points:** 31.8% (n=47)	20.8% (n=5)	36.4% (n=4)	36.0% (n=9)	21.2% (n=14)	66.7% (n=14)
➢ **1 point:** 37.2% (n=55)	29.2% (n=7)	27.3% (n=3)	20.0% (n=5)	53.0% (n=35)	23.8% (n=5)
➢ **2 points**: 14.9% (n=22)	20.8% (n=5)	18.2% (n=2)	12.0% (n=3)	16.7% (n=11)	4.8% (n=1)
➢ **3 points:** 11.5% (n=17)	20.8% (n=5)	9.1% (n=1)	24.0% (n=6)	7.6% (n=5)	-
➢ **4 points:** 3.4% (n=5)	4.2% (n=1)	9.1% (n=1)	8.0% (n=2)	-	4.8% (n=1)
➢ **5 points:** 0.7% (n=1)	-	-	-	1.5% (n=1)	-
➢ **6 points:** 0.7% (n=1)	4.2% (n=1)	-	-	-	-
**Diagnostic results from consultation**					
**Total IgE antibodies [U/ml]:** (n=143 a.d.)<br>M 27.0; Ø 92.9 ± 185.4<br>R: <10-1090<br>➢ **> 100 U/ml:** 20.3% (n=30)	(n=22 a.d.)<br>M 61.8; Ø 145.7<br>R: <10-780<br>37.5% (n=9)	M 49.0; Ø 161.6<br>R: <10-1085;<br>27.3% (n=3)	M 40.0; Ø 138.2<br>R: <10-1090;<br>24.0% (n=6)	M 20.0; Ø 63.5<br>R: <10-820;<br>15.2% (n=10)	(n=18 a.d.)<br>M 17.0; Ø 29.2<br>R: <10-129;<br>4.8% (n=1)

Parameter: Overall collective (n=148)	*Group 1 (n=24) confirmed*	*Group 2 (n=11) likely*	*Group 3 (n=25) unclear*	*Group 4 (n=66) unlikely*	*Group 5 (n=21) no causation*
➢ **> 150 U/ml:** 13.5% (n=20)	29.2% (n=7)	18.2% (n=2)	16.0% (n=4)	10.6% (n=7)	-
➢ **> 400 U/ml:** 5.4% (n=8) (2x with blood eosinophilia)	8.3% (n=2)	9.1% (n=1)	12.0% (n=3)	3.0% (n=3)	-
**Baseline Bodyplethysmography** (n=147 a.d.)			(n=24 a.d.)		
➢ **current airway obstruction**: 19.6% (n=29)	20.8% (n=5)	36.4% (n=4)	12.0% (n=3)	27.3% (n=18)	-
➢ **borderline airway obstruction**: 4.7% (n=7)	4.2% (n=1)	-	8.0% (n=2)	6.1% (n=4)	-
➢ **pulmonary overinflation**: 13.5% (n=20)	16.7% (n=4)	9.1% (n=1)	4.0% (n=1)	15.2% (n=10)	19.0% (n=4)
➢ **borderline pulmonary overinflation**: 6.1% (n=9)	8.3% (n=2)	18.2% (n=2)	8.0% (n=2)	3.0% (n=2)	4.8% (n=1)
➢ **pulmonary restriction:** 0.7% (n=1)	-	-	4.0% (n=1)	-	-
**Metacholine challenge/MCT:** performed in n=114 (77%)	n=17 (71%)	n=6 (55%)	n=20 (80%)	n=52 (79%)	n=18 (86%)
➢ **BH proven:** 33.8% (n=50)	33.3% (n=8)	36.4% (n=4)	48.0% (n=12)	37.9% (n=25)	-
➢ **borderline BH:** 4.7% (n=7)	-	9.1% (n=1)	4.0% (n=1)	4.5% (n=3)	9.5% (n=2)
➢ **no BH proven or BH highly unlikely:** 31.1% (n=46)	33.3% (n=8)	9.1% (n=1)	20.0% (n=5)	28.8% (n=19)	61.9% (n=13)
➢ **discontinued early => not conclusive**: 7.4% (n=11)	4.2% (n=1)	-	8.0% (n=2)	7.6% (n=5)	14.3% (n=3)
**Synopsis from baseline bodyplethysmography and MCT at consultation**					
➢ **Confirmed OVD**: 48.6% (n=72)	50.0% (n=12)	63.6% (n=7)	52.0% (n=13)	59.1% (n=39)	-
➢ **Questionable OVD:** 5.4% (n=8)	-	9.1% (n=1)	4.0% (n=1)	6.1% (n=4)	9.5% (n=2)
➢ **Currently not enough OVD evidence**: 27.0% (n=40)	29.2% (n=7)	9.1% (n=1)	20.0% (n=5)	21.2% (n=14)	61.9% (n=13)
➢ **Tests insufficient to rule for or against OVD**: 18.9% (n=28)	20.8% (n=5)	18.2% (n=2)	24.0% (n=6)	13.6% (n=9)	28.6% (n=6)
**Evaluable SPT (n=136 with sufficient controls) for:**					
➢ **environmental inhalation allergens (EA)**: tested in n=132	n=22	n=7	n=18	n=65	n=19
➢ **positive SPT reaction(s):** 59.1% (n=78) of EA tests (= 52.7% in overall collective)	81.8% (n=18) EA tests	57.1% (n=4) EA tests	77.8% (n=14) EA tests	60.0% (n=39) EA tests	15.8% (n=3) EA tests
➢ **only questionable reaction(s):** 6.8% (n=9) of EA tests	-	-	11.1% (n=2) EA tests	3.1% (n=2) EA tests	26.3% (n=5) EA tests
➢ **latex**: tested in n=35			n=4	n=19	n=4
➢ **positive reaction**: 2.9% (n=1) of latex tests	n=6	n=1	-	-	-
➢ **questionable reaction**: 11.4% (n=4) of latex tests	16.6% (n=1) latex	-	25% (n=1) latex	5.3% (n=1) latex	-
➢ **henna:** tested in n=22	33.3% (n=2) latex	-	n=1	n=11	n=6

Parameter: Overall collective (n=148)	*Group 1 (n=24) confirmed*	*Group 2 (n=11) likely*	*Group 3 (n=25) unclear*	*Group 4 (n=66) unlikely*	*Group 5 (n=21) no causation*
➢ **positive reaction**: 9.1% (n=2) of henna tests	n=2	n=2	-	-	17% (n=1) henna
➢ **Ammonium persulfate (AP) (valid tests)**: tested in n=134	-<br>n=22	50% (n=1) henna<br>n=7	n=18	n=66	n=21
➢ **positive reaction (wheal ≥ 3mm):** 15.7% (n=21) of AP tests	86.3% (n=19) of AP tests	14.3% (n=1) of AP tests	5.6% (n=1) of AP tests	-	-
➢ **questionable reaction (wheal 1-2mm):** 6.7% (n=9) of AP tests	4.5% (n=1) of AP tests	42.9% (n=3) of AP tests	16.7% (n=3) of AP tests	3.0% (n=2) of AP tests	-
➢ **other own hairdyes**: tested in n=8 (7x combined with AP	n=2<br>50% (n=1) of tests	-<br>-	n=4<br>-	n=3<br>-	n=1<br>-
➢ **positive reaction**: 12.5% (n=1) of tests					
(positive reaction ≠ equivalent to clinical relevance)					
**Specific inhalation challenge / SIC:** 6.1% (n=9)	12.5% (n=3)	-	4.0% (n=1)	6.1% (n=4)	4.8% (n=1)
➢ **positive SIC reaction:** 2.0% (n=3), (33% of SIC)	12.5% (n=3)	-	-	-	-
**Previous medical findings relevant for case evaluation**					
**Previous skin tests to identify immediate-type hyper-sensitivity reaction to AP or blonde dyes**: 6.1% (n=9)	16.7% (n=4)	18.2% (n=2)	-	1.5% (n=1)	9.5% (n=2)
➢ **Reported positive reaction**: 5.4% (n=8) (test quality and clinical relevance not considered)	16.7% (n=4)	18.2% (n=2)	-	1.5% (n=1)	4.8% (n=1)
➢ **Considering test quality and relevance in synopsis with other findings (SPT, SIC), cases/tests rated as**					
➢ **positive / clinically relevant:** 2.7% (n=4) *	16.7% (n=4)	-	-	-	-
➢ **possible / relevance likely**: 0.7% (n=1)	-	9.1% (n=1)	-	-	-
➢ **negative**: 1.4% (n=2) (2x SPT & SIC negative)	-	-	-	1.5% (n=1)	4.8% (n=1)
➢ **not appraisable/relevance unclear**: 0.7% (n=1)	-	9.1% (n=1)	-	-	-
*(2x adaquately quality-controlled, 1x SPT at consultation also pos., 1x SIC pos.)					
**Previous lung function tests showing OVD**: 26.3% (n=39)	25.0% (n=6)	9.1% (n=1)	36.0% (n=9)	34.8% (n=23)	-
**Previous positive SPT or known type-I-sensitization against EA:** 45.9% (n=68)	54.2% (n=13)	54.5% (n=6)	56.0% (n=14)	47.0% (n=31)	19.0% (n=4)

Parameter: Overall collective (n=148)	*Group 1 (n=24) confirmed*	*Group 2 (n=11) likely*	*Group 3 (n=25) unclear*	*Group 4 (n=66) unlikely*	*Group 5 (n=21) no causation*
**Summary medical rating (previous external findings and consultation together)**					
**Obstructive lower airway disease**					
➢ **confirmed:** 60.1% (n=89)	62.5% (n=15)	72.7% (n=8)	72.0% (n=18)	71.2% (n=47)	-
➢ **possible, but not verified**: 24.3% (n=36)	25.0% (n=6)	18.2% (n=2)	24.0% (n=6)	12.1% (n=8)	66.7% (n=14)
➢ **unlikely**: 15.5% (n=23)	12.5% (n=3)	9.1% (n=1)	4.0% (n=1)	16.7% (n=11)	33.3% (n=7)
**Immediate-type-like specific hypersensitivity reaction to hairdresser's material**					
➢ **AP or blonde dye**					
➢ clinically relevant: 14.9% (n=22)	91.7% (n=22)	-	-	-	-
➢ questionable: 3.4% (n=5)	-	45.5% (n=5)	-	-	-
➢ not appraisable: 9.5% (n=14)	8.3% (n=2)	36.4% (n=4)	28.0% (n=7)	-	-
➢ no reaction or no clinical relevance: 72.3% (n=107)	-	18.2% (n=2)	72.0% (n=18)	100% (n=66)	100% (n=21)
➢ **henna**					
➢ clinically relevant: 0.7% (n=1)	-	9.1% (n=1)	-	-	-
**Type-I-sensitization to EA**: 62.6% (n=92)	83.3% (n=20)	54.5% (n=6)	72.0% (n=18)	65.2% (n=43)	23.8% (n=5)
➢ rated as clinically relevant (in synopsis with anamnesis for allergic RCA and/or asthma): 41.2% (n=61) overall (66% of EA sensitizations)	41.7% (n=10)	45.5% (n=5)	52.0% (n=13)	43.9% (n=29)	19.0% (n=4)

(Group 1: occupational causation confirmed; Group 2: occupational causation likely; Group 3: occupational causation unclear; Group 4: occupational causation unlikely; Group 5: no occupational causation). In one case hairdressing was not the focus of the medical consultation and it was therefore left out of the subgroup classification. To avoid data bias, no statistic parameters (median, mean) were calculated (n.c.) for subgroups when data were available for no more than half of the collective. Percentages and descriptive statistics given are based on the whole collective (n=148) or the respective subgroup size, when not otherwise specified.

(a.d.: available data; AP: ammonium persulfate; BH: bronchial hyperreactivity; BMI: Body mass index; EA: Environmental inhalation allergen; M: median; Ø: arithmetic mean; MCT: metacholin challenge test; n.c.: not calculated; ot: over time; OVD: obstructive ventilation

disorder; R: range; RCA: rhinoconjunctivitis allergica; SADS: shortend atopic diathesis score; SIC: Specific inhalation challenge; SPT: skin prick test)
