# Supplementary material for: Respiratory afflictions during hairdressing jobs: case history and clinical evaluation of a large symptomatic case series
Source: J Occup Med Toxicol. 2022 May 23;17:10. doi: 10.1186/s12995-022-00351-5 (PMC9125837; doi:10.1186/s12995-022-00351-5)
Supplement: Supplementary file 4 — Additional file 4. Heat map – visual illustration of the magnitude of subgroup variations. [file 12995_2022_351_MOESM4_ESM.pdf]

<u>Additional file 4:</u> Heat map - visual illustration of the magnitude of subgroup variations

Visual display of variations* between causation subgroups (G1-5) and the overall data in a heat map for selected personal and medical parameters of a symptomatic hairdressers' collective (n=148) with a history of respiratory symptoms at work.

Based on	Parameter	overall	G 1	G 2	G 3	G 4	G 5
Demographics / General medical data							
% of cases with	Female sex	91.9					
Ø of	Age at consultation [years]	39.7					
Ø of	BMI [kg/m²]	25.5					
% of cases with	BMI > 30kg/m²	16.2					
% of	Never smoker	39.9					
% of	Ex-smoker	40.5					
% of	Current smoker	18.2					
% of cases with	Antiobstructive medication	74.3					
% of cases with	permanent antiobstructive medication	58.8					
% of cases with	Antiobstructive medication active during lung function testing	58.1					
% of cases with	private pet contact	39.2					
Occupational anamnesis / Working conditions							
Ø of	Duration of symptoms at work [years]	5.5					
Ø of	Age at initial symptoms at work [years]	34.0					
Ø of	Hairdresser tenure at initial symptoms [years]	15.0					
% of cases with	Change of job	23.6					
% of cases with	On sick-leave ≥ 2 month	6.8					
% of cases with	Currently self-employed hairdresser	23.0					
% of cases with	Full-time work (≥ 35h/week)	40.5					
% of cases with	History of mold at workplace	6.1					
Ø of	Number of customer seats in service [n]	7.1					
% of cases with	Separated room for dye preparing	26.4					

Based on	Parameter	overall	G 1	G 2	G 3	G 4	G 5
% of cases with	Use of gloves at work	70.2					
% of cases with	Regular fresh air at saloon	61.5					
% of cases with	Technical ventilation / air conditioning	23.0					
% of cases with	Symptoms progression from exclusive upper airways initially to lower airways over time	16.9					
% of cases with	Start with concurrent bronchial infect	13.5					
% of cases with	Seasonal influence / fluctuation	24.3					
% of cases with	Latency: immediate airway symptoms within 10 min of daily exposure start	73.4					
% of cases with	Latency: more than 1 h between daily exposure start and airway symptoms	7.6					
% of cases with	Improvement **only** during vacation/longer time off work	23.0					
% of cases with	Symptoms improvement during weekends:	48.6					
% of cases with	Workplace trigger: Stay in saloon in general	5.4					
% of cases with	Specific workplace triggers named (multiple answers possible)	93.2					
% of cases with	Specific workplace triggers: hair dyes in general	83.1					
% of cases with	Specific workplace triggers: blonde dyes	81.1					
% of cases with	Specific workplace triggers: hair spray	60.1					
% of cases with	Specific workplace triggers: permanent wave	35.8					
% of cases with	Work-related urticaria in contact with hair/blonde dyes or AP	9.5					
% of cases with	Urticarial skin afflictions already at symptoms start	6.8					
% of cases with	History of work-related hand eczema	36.5					
% of cases with	Current hand eczema at consultation	8.1					
% of cases with	Known contact sensitization to hairdressers substance(s) overall	21.6					
% of cases with	Known contact sensitization to AP	8.1					
% of hand eczema cases with	Known contact sensitization to hairdressers substance(s) in cases	33.3					
% of cases with	Dust, fume, vapor, odour as unspecific triggers for airway symptoms	68.2					
% of cases with	Stress in general as unspecific trigger for airway symptoms	24.3					
% of cases with	Aggravation by airway infect as unspecific trigger for airway symptoms	20.3					
% of cases with	Ubiquitous inhalation allergen as unspecific triggers for airway symptoms	11.5					
Other medical conditions / Diagnostic results							
% of cases with	Known hypertonus	14.9					

Based on	Parameter	overall	G 1	G 2	G 3	G 4	G 5
% of cases with	Recurrent or chronic sinusitis or s/p sinus surgery	24.3					
% of cases with	Nasal polyps (or s/p polyp surgery)	10.8					
% of cases with	Abnormalities of the nasal concha or septum or s/p surgery	9.4					
% of cases with	Reflux oesophagitis	24.3					
% of cases with	Known thyroid issue (s/p, hyper- or hypofunction)	21.6					
% of cases with	Allergic rhinoconjunctivitis to ubiquitous inhalation allergens	39.1					
% of cases with	Allergic asthma bronchiale to ubiquitous inhalation allergens	8.8					
% of cases with	(History of) atopic dermatitis	8.8					
% of cases with	Positive family anamnesis for atopic diseases (1° relatives)	37.8					
Ø of	Shortened atopic diathesis score (SADS) *(0 = lowest score, 7 = highest)*	1.22					
% of cases with	0 points in SADS	31.80					
% of cases with	1 point in SADS	37.2					
% of cases with	2 points in SADS	14.9					
% of cases with	3 points in SADS	11.5					
Ø of	Total IgE antibodies [U/mL]	92.9					
% of cases with	IgE > 100 U/mL	20.3					
% of cases with	IgE > 150 U/mL	13.5					
% of cases with	IgE > 400 U/mL	5.4					
% of EA SPT tests	Only questionable SPT reaction(s) to EA	6.8					
% of EA SPT tests	Positive skin prick test (SPT) reaction(s) to environmental inhalation allergens (EA)	59.1					
% of cases with	Previous positive SPT or known type-I-sensitization against EA reported	45.9					
% of cases with	Type-I-sensitization to EA (external findings and current SPT result combined)	62.6					
% of cases with	Type-I-sensitization to EA rated as clinically relevant (in synopsis with anamnesis)	41.2					

*Downward deviation from the baseline percentage or arithmetic mean of the overall collective (=white) is displayed by blue coloring, upward deviation by red. The upper and lower limit of the coloring scale was based on the respective parameter and set at ±34.1% (in case of percentages) respectively ±standard deviation (in case of arithmetic mean). Parameters where differences among subgroups

are expected a priori due to subgroup definitions (e.g. lung function testing, final airway diagnosis, skin prick tests of working materials) or occurrence in the overall collective is under 5% are not depicted.

(*AP: ammonium persulfate; BH: bronchial hyperreactivity; Ø: arithmetic mean; MCT: metacholin challenge test, OVD: obstructive ventilation disorder; SIC: Specific inhalation challenge; SPT: skin prick test*)
