# Supplementary material for: Respiratory afflictions during hairdressing jobs: case history and clinical evaluation of a large symptomatic case series
Source: J Occup Med Toxicol. 2022 May 23;17:10. doi: 10.1186/s12995-022-00351-5 (PMC9125837; doi:10.1186/s12995-022-00351-5)

<u>Additional file 5:</u> Association between age and tenure time at initial symptoms (Supplementary figure)

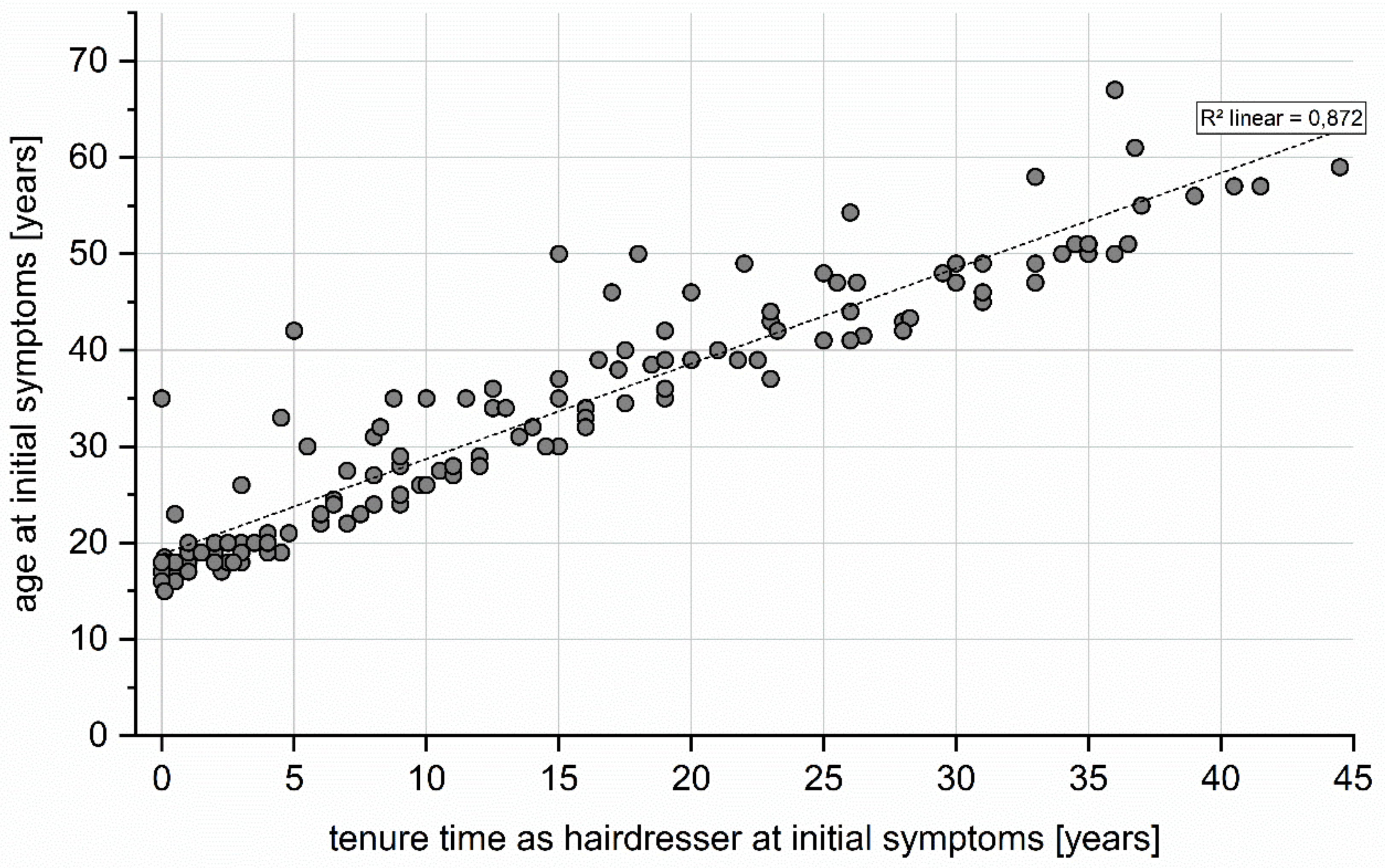

Supplement: Supplementary file 5 — Additional file 5. Association between age and tenure time at initial symptoms (supplementary figure). [file 12995_2022_351_MOESM5_ESM.pdf]
