# Supplementary material for: Respiratory afflictions during hairdressing jobs: case history and clinical evaluation of a large symptomatic case series
Source: J Occup Med Toxicol. 2022 May 23;17:10. doi: 10.1186/s12995-022-00351-5 (PMC9125837; doi:10.1186/s12995-022-00351-5)

Hiller, Greiner, Drexler. Respiratory afflictions during hairdressing jobs: Case history and clinical evaluation of a large symptomatic case series.

Additional file 6: Age and tenure time at initial symptoms overall and broken down by group-wise depiction of those parameters among case classification for specific immunological occupational causation (supplementary figure)

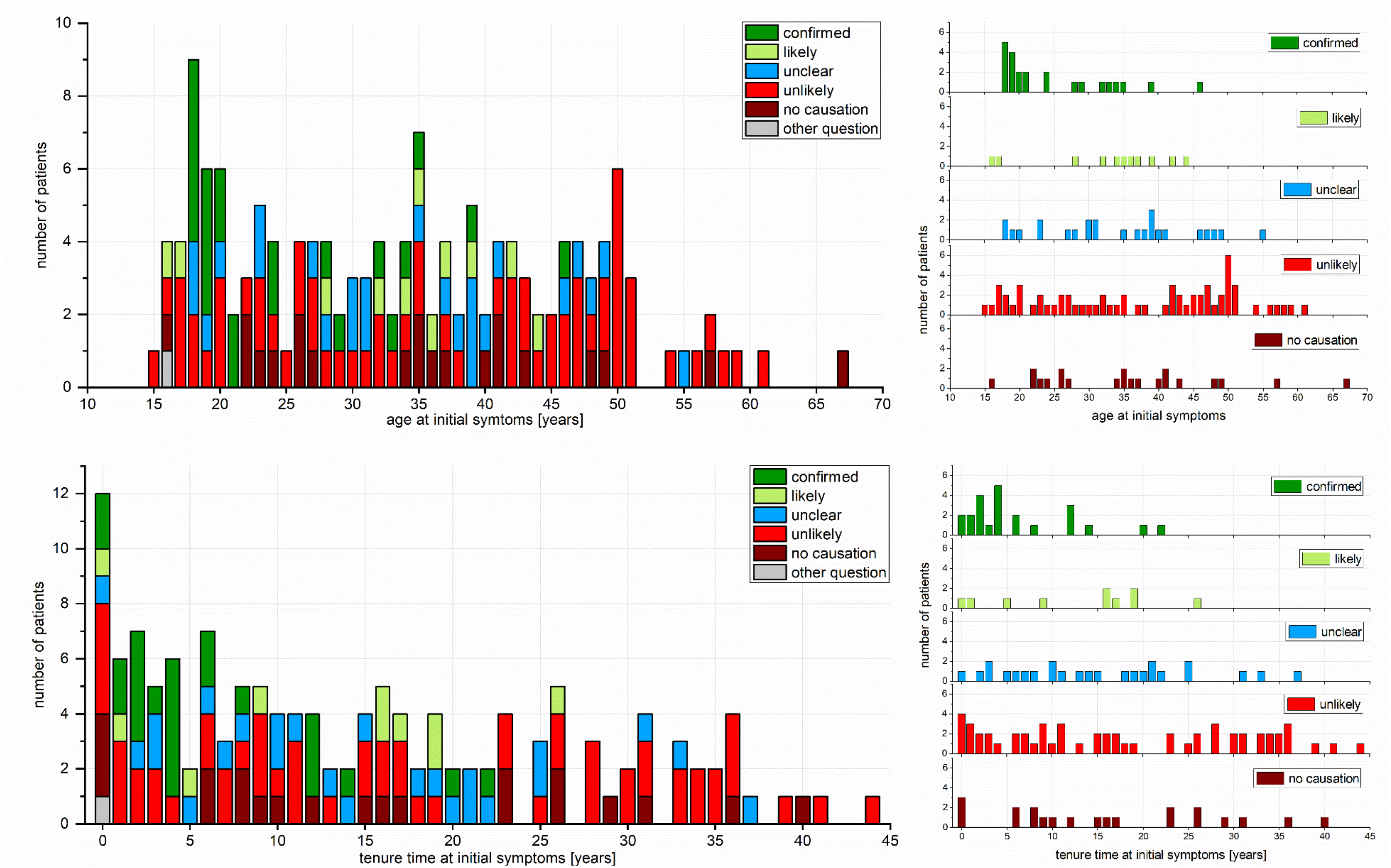

Supplement: Supplementary file 6 — Additional file 6. Age and tenure time at initial symptoms overall and broken down by group-wise depiction of those parameters among case classification for specific immunological occupational causation (supplementary figure). [file 12995_2022_351_MOESM6_ESM.pdf]
